# Non-shivering thermogenesis is differentially regulated during the hibernation season in Arctic ground squirrels

**DOI:** 10.3389/fphys.2023.1207529

**Published:** 2023-07-13

**Authors:** Moriah Hunstiger, Michelle Marie Johannsen, S. Ryan Oliver

**Affiliations:** ^1^ Department of Chemistry and Biochemistry, University of Alaska Fairbanks, Fairbanks, AK, United States; ^2^ Institute of Arctic Biology, University of Alaska Fairbanks, Fairbanks, AK, United States

**Keywords:** hibernation, sarcolipin, ground squirrel, non-shivering thermogenesis, metabolism

## Abstract

Arctic ground squirrels are small mammals that experience physiological extremes during the hibernation season. Body temperature rises from 1°C to 40°C during interbout arousal and requires tight thermoregulation to maintain rheostasis. Tissues from wild-caught Arctic ground squirrels were sampled over 9 months to assess the expression of proteins key to thermogenic regulation. Animals were sacrificed while aroused, and the extensor digitorum longus, diaphragm, brown adipose tissue, and white adipose tissue were probed using Western blots to assess protein expression and blood was sampled for metabolite analysis. Significant seasonal expression patterns emerged showing differential regulation. Contrary to our prediction, white adipose tissue showed no expression of uncoupling protein 1, but utilization of uncoupling protein 1 peaked in brown adipose tissue during the winter months and began to taper after terminal arousal in the spring. The opposite was true for muscular non-shivering thermogenesis. Sarco/endoplasmic reticulum calcium ATPase 1a and 2a expressions were depressed during the late hibernation season and rebounded after terminal arousal in diaphragm tissues, but only SERCA2a was differentially expressed in the extensor digitorum longus. The uncoupler, sarcolipin, was only detected in diaphragm samples and had a decreased expression during hibernation. The differential timing of these non-shivering pathways indicated distinct functions in maintaining thermogenesis which may depend on burrow temperature, availability of endogenous resources, and other seasonal activity demands on these tissues. These results could be impacted by fiber type makeup of the muscles collected, the body weight of the animal, and the date of entrance or exit from hibernation.

## Introduction

Among mammals, there are many strategies to overcome the environmental challenges of food scarcity and harsh thermal conditions. One of these strategies is hibernation, a physiological phenomenon whereby an animal deviates from the mammalian pattern of homeothermy to that of heterothermy (Heldmaier et al., 2004). A distinct hallmark of homeothermy is a consistently high body temperature, in the range between 34 and 42°C for most mammals (Ivanov, 2006; Porter & Kearney, 2009). The heterothermy phenotype is characterized by periods of suppressed metabolism, which consequently reduces metabolic heat production and can present widely from short daily variations to seasonal multiday decreases in body temperature depending on the species (Grigg et al., 2004). Mammals that experience heterothermy include members from all three subclasses of mammals, namely, monotremes, marsupials, and eutherians. Among the eutherians are bears, bats, hedgehogs, hamsters, tenrecs, and ground squirrels (Grabek et al., 2015). An extreme example of heterothermy is the hibernation phenotype expressed by the Arctic ground squirrel (AGS), *Urocitellus parryii.*

Hibernation in AGS is characterized by periods of torpor during the fall and winter months and a return to homeothermy during spring and summer (Buck & Barnes, 1999; Barnes & Ritter, 2019). Torpid AGSs undergo significantly reduced metabolic rates and concomitant reduction in body temperature (Buck & Barnes, 1999; Barnes & Ritter, 2019). However, torpor is not a physiological state that remains constant across the hibernation season but occurs in 2- to 3-week bouts interrupted by interbout arousals (IBAs)—discrete intervals when the metabolic rate and body temperature return to euthermic levels (Buck et al., 2008; Karpovich et al., 2009). During an IBA, AGSs revert from a state of low metabolism to a state of high-energy expenditure in a relatively short period of time, between 10 and 20 h for a typical mid-winter IBA (Buck et al., 2008). Rewarming, during an IBA, can cause metabolic rates that are six times greater than the resting metabolic rate and has two distinct phases with the first phase initiated by non-shivering thermogenesis (NST) in brown adipose tissue (BAT) until the body temperature is approximately 16°C (Tøien et al., 2001; Karpovich et al., 2009; Williams et al., 2011). Shivering is recruited during the second phase of an IBA to increase the body temperature until euthermic levels are reached (Tøien et al., 2001; Cannon & Nedergaard, 2004; Williams et al., 2011). During the initial rewarming phase of an IBA, the AGS's body temperature increases from a torpid body temperature of approximately 1°C to approximately 40°C (Buck & Barnes, 1999; Barnes & Ritter, 2019). This discrepancy in the body temperature results in IBAs being energetically expensive, and it is estimated that arousals account for up to 86% of energy expenditure during a complete torpor–arousal cycle in the AGS hibernating at an environmental temperature of 2°C (Karpovich et al., 2009). Because rewarming and cooling during a full IBA cycle is so energetically costly, it requires tight regulation to rapidly increase and decrease the body temperature to ensure metabolic rheostasis.

The use of uncoupling protein 1 (UCP1) in BAT is a well-documented mechanism for heat production (NST) in small eutherians (Cannon & Nedergaard, 2004). Heat is generated when UCP1 allows protons to leak back across the mitochondrial inner membrane, dissipating the proton motive force created by the electron transport chain (Cannon & Nedergaard, 2004). The presence of UCP1 uncoupling is thought to increase the capacity by 20% to generate heat in BAT (Cannon & Nedergaard, 2004). By comparison, beige or brite adipocytes are induced thermogenic cells in white adipose tissue (WAT) that can be adapted by chronic cold exposure or other environmental factors to mimic the properties of brown adipocytes and utilize UCP1 (Cannon & Nedergaard, 2004; Wu et al., 2012; Ikeda & Yamada, 2020). Brite cells can contribute to NST using UCP1 and can also use calcium-cycling via sarco/endoplasmic reticulum calcium ATPase 2b (SERCA2b) to facilitate rewarming mechanisms (Cannon & Nedergaard, 2004; Wu et al., 2012; Ikeda & Yamada, 2020). By increasing the amount of cold-induced thermogenic tissue or the concentration of thermogenic proteins, an animal can greatly increase the capacity for NST, metabolism, and temperature regulation (Cannon & Nedergaard, 2004; Wu et al., 2012; Ikeda & Yamada, 2020).

Recently, a new mechanism of NST has been proposed in which sarcolipin (SLN), and possibly phospholamban (PLB), uncouples SERCA1a/2a ATP hydrolysis in skeletal muscle tissues. The proposed mechanism, similar to UCP1 in fat tissues, suggests that heat is produced when SLN uncouples the hydrolysis of ATP from the calcium pumping action of SERCA (Pant et al., 2016). SLN is widely expressed in neonatal mouse skeletal muscle but that expression decreases in fast-twitch muscles like the extensor digitorum longus (EDL) as development progresses but the expression of SLN remains high in fast-oxidative and slow-twitch muscles like the diaphragm (DIA) (Rowland, Bal, & Periasamy, 2015a; Pant et al., 2015; Sopariwala et al., 2015; Bal et al., 2021). However, the expression of SLN in neonatal mice in cold acclimatized conditions increases in fast-twitch muscles (Pant et al., 2015; Bal et al., 2021). This indicates that depending on the fiber type composition, muscles will express SLN in response to cold stress supporting the postulation that the induction of SLN uncoupling may be involved in thermogenic regulation (Bal et al., 2021). This mechanism is additionally supported when mice with reduced BAT thermogenesis capabilities can maintain the core body temperature in the presence of cold challenge, indicating some sort of compensatory metabolic process (Bal et al., 2012; 2017). In a partner study, when SLN^−/−^ mice were exposed to severe cold (4°C), there was a drastic decrease in the core body temperature from 37°C to 32.2°C ± 1.4°C after 4 h and a precipitous drop to 26.9°C ± 1.9°C after 6 h (Bal et al., 2012; 2017), indicating that SLN uncoupling could be a contributor to the overall metabolism and heat production. This mechanism has demonstrated capabilities to potentially compensate for the lack of UCP1 thermogenesis when mice with ablated interscapular BAT defended their body temperature, 35.8°C ± 0.4°C, similar to their counterparts with intact BAT, 36.3°C ± 0.2°C (Bal et al., 2012). Additionally, when UCP1:SLN double knockout mice or SLN^−/−^ mice with downregulated activity of BAT were challenged with acute cold, hypothermia was induced, and in gradual cold conditions, mice could not maintain adequate fat stores (Rowland et al., 2015a; Bal et al., 2017). Taken together, these studies indicate that both UCP1 and SLN are essential for optimal thermogenesis in mice and other small mammals.

The most studied animal models are currently eutherian homeotherms that maintain a relatively stable body temperature when compared to their heterothermic counterparts. We propose that NST in the skeletal muscles via SLN/SERCA uncoupling could be a mechanism employed by AGSs, in conjunction with thermogenic adipose tissue and shivering, to assist both in metabolism and large temperature ranges experienced by AGSs across the hibernation season as well as in acute conditions of an IBA. This project provides a valuable comparison between homeotherms and heterotherms to better understand the capacity of these uncouplers contributing to thermogenesis during times of increased metabolic stress. This research seeks to identify if these proteins are expressed in AGS, they vary by muscle group based on the fiber type makeup or muscle activity, and how the expression patterns of these proteins during hibernation can give insight into how these thermogenic mechanisms contribute to energy utilization and metabolic regulation in this novel hibernator model. It was hypothesized that SLN/SERCA uncoupling and UCP1 uncoupling would follow similar increased expression patterns within their respective tissues—muscle and adipose—during early and late hibernation IBAs when compared to pre- and post-hibernation samples, with the muscle groups likely varying in expression patterns. Specifically, EDL and DIA in AGSs will both express proteins that are involved in muscular NST, but they may show varied expressions based on the fiber type makeup.

## Methods

### Animals

AGSs (*U. parryii*) were wild caught in the Brooks Range near Toolik Field Station of the University of Alaska Fairbanks (UAF) during July 2016 under a permit from the Alaska Department of Fish and Game. Upon being taken to the UAF animal facility, the animals were moved to single metal cages and screened for *Salmonella*, and they underwent a 2-week-long quarantine. The animals were initially housed under simulated summer conditions of 20°C with a 12 h:12 h light:dark cycle. During the transition out of the summer active season, August and early September, the animals were moved to a cold chamber at 2°C with a 4 h:20 h light:dark cycle to simulate winter conditions. The animals were daily checked, and water bottles were filled to provide water *ad libitum*. A total of 10 pellets of Mazuri rodent chow were provided daily to the animals until signs of hibernation were observed. Hibernating animals were visually assessed for respiration, body posture, and behavior parameters for indications of hibernation status. Hibernating AGSs typically adopt a curled position within the nest with their backs oriented upward and tails tucked under their abdomen and chest. Breathing decreases to 1–2 shallow breaths per minute, and they have a depressed body temperature of 0–2°C. Hibernation was communicated to the staff and researchers by placing wood shavings on the animal’s back, and if the shavings were undisturbed for 24 h, then the animal was determined to be hibernating.

All animal procedures were reviewed and approved by the Animal Care and Use Committee of the University of Alaska Fairbanks (IACUC #864841) and were consistent with the guidelines established by the veterinary staff and the UAF animal facility.

### Tissue collection and processing

**FIGURE 1:**
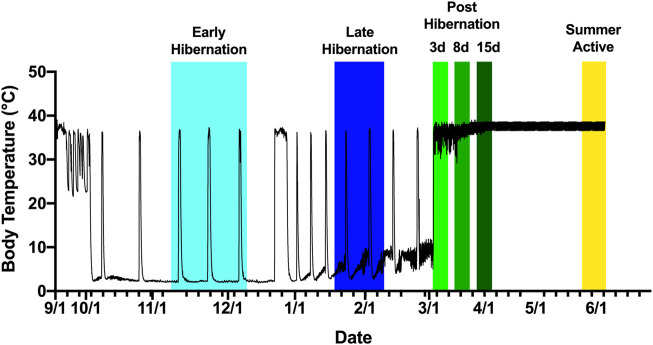
Tissue collection scheme based on body temperature of an Arctic ground squirrel during hibernation. Internal body temperature during hibernation season is shown as a solid black line. Animals were sampled aroused or active from November 2016 to June 2017. Early hibernation was defined as undergoing at least 2–3 full torpor bouts shown in light blue ranging from 15 November to 8 December. Late hibernation was defined as continued regular torpor bouts after the turn of the new year, with IBAs shown in dark blue ranging from 22 January to 18 February. Post hibernation (days 3,8, and 15) defined as arousal from torpor without re-entrance into torpor shown in shades of green ranging from 28 February to 3 April. Summer active defined as a return to euthermic phenotype but still exposed to cold shown in yellow ranging from 3 June to 13 June.

Euthanasia and opportunistic tissue sample collection occurred at six distinct time points, namely, cold exposed summer active, early hibernation season, late hibernation season, 3 days post arousal, 8 days post terminal arousal, 15 days post terminal arousal, and cold exposed summer active with all groups having n = 4 based on an *a priori* power analysis with a power of 0.9, error of 0.05, and hibernation rate of 100% (Figure 1). Summer active samples were collected in early June after terminal arousal in April and May when the animals stopped expressing torpor and returned to normothermic body temperatures (Figure 1). All animals had their rectal body temperature measured prior to tissue collection. Only tissues from animals with a body temperature greater than 34°C were used in this study. AGSs in the summer cohort were still housed at winter temperatures, 2°C, but had transitioned to summer light conditions. During early and late hibernation, samples were collected during an IBA when thermogenic proteins were predicted to be highly expressed based on the assertion that slow loss of protein integrity during torpor and the resultant need for replenishment may provide the impetus for arousal episodes (Epperson et al., 2004; Martin et al., 2008). Five tissues were collected from each animal, namely, blood, DIA, EDL, WAT, and BAT. The muscles were chosen based on fiber type specificity in other rodents. The DIA is constitutively active during torpor, and in other rodent species has been shown to have a mixed fiber type makeup (Eddinger et al., 1985). The EDL in mice and rats is dominant in Type II muscle fibers (Eddinger et al., 1985; Soukup et al., 2002; Schiaffino & Reggiani, 2011). The blood was centrifuged (chilled) at 3,000 *g* for 10 min and plasma was removed and frozen in liquid nitrogen. The muscle tissues were flash-frozen, in foil packets, using liquid nitrogen, and placed in a −80°C freezer for later analysis.

The whole frozen muscle tissue was placed in a falcon tube with 2–5 mL homogenizing buffer (10 mM sodium bicarbonate, 2 mM sodium azide, 10 mM tris-Cl, and pH 7.5) in a volume relative to tissue mass (range: 0.2–0.79 g in 2 mL, 0.8–1.59 g in 3 mL, 1.6–2.39 g in 4 mL, and 2.4 or greater in 5 mL). The tissue was finely minced with scissors and subjected to three rounds of 15 s homogenization using a Tekmar Tissuemizer, resting on ice between rounds. The homogenate was filtered through two layers of gauze to remove fascia, tendons, or large unhomogenized tissue pieces. The homogenizing tube was rinsed onto a gauze with 1 mL homogenizing buffer to minimize the loss of sample. The filtered homogenate was centrifuged at 2,000 *g* for 10 min. The supernatant was removed and centrifuged at 10,000 *g* for 5 min (Fisher Scientific accuSpin Micro 17R). Then, the supernatant was gently agitated on a refrigerated shaker with KCl to solubilize myosin globules for 30 min. The samples were then centrifuged at 40,000 *g* for 15 min (Beckman Coulter Allegra 64R Centrifuge). The supernatant was removed and sarcoplasmic reticulum pellets were suspended in 200 µL lysis buffer (150 mM sodium chloride, 5 mM EDTA, 50 mM Tris, 1.0% Triton-X, 10% sodium deoxycholate, 10% SDS, and pH 8) and mixed using repeated pipetting. Protein quantification was performed using the Modified Lowry Protein Assay Kit (Thermo Scientific 23240), and the samples were stored at −80°C until later analysis.

Adipose tissue was extracted using the Minute Total Protein Extraction Kit for adipose tissue/cultured adipocytes (Invent Biotechnologies AT-022) where 50–80 mg portions of tissues were homogenized with extraction powder and buffer A via mortar and pestle. Slurry was then centrifuged, frozen to solidify excess adipose residues, and then centrifuged again. The resultant supernatants received approximately 20 μL of buffer B, with brown adipose extracts being additionally diluted up to 1 mL with DI water. The total protein concentration was calculated using the filter paper assay method by blotting 1 μL of BAT extract or 3 μL of WAT extract and BSA standards (0–8 μg) on a filter paper, and proteins were stained using Coomassie brilliant blue G stain (Minamide & Bamburg, 1990). Dye was extracted and absorbance was measured at 600 nm in a 96-well plate, and the protein concentration was calculated based on absorbance values from the bovine serum albumin (BSA) standard curve with resulting concentrations falling between 1.5 and 10 μ/μL (Minamide & Bamburg, 1990). The samples were then stored at −80°C.

### Western blots

The Western blot analysis was performed to determine the expression levels of SLN, PLB, UCP1, SERCA1a, and SERCA2a in samples. Proteins from tissue homogenates were separated in tricine-based SDS-PAGE for small proteins with 10 µg protein loaded (4% stacking and 16% resolving for PLB and SLN) and were run at 20 V for 45 min and then 90 V until the dye front had migrated 75% of the way down the gel. Standard tris-glycine SDS-PAGE was used for larger proteins with 5 µg protein loaded (4% stacking and 12% resolving for SERCA1a, SERCA2a, and UCP1) and was run at 190 V until the dye front reached near the bottom of the gel. Separated proteins were transferred onto 0.2 µm nitrocellulose at 75 V for 3 h using a wet transfer method. After transfer, the membranes were rinsed for 5 min using a Ponceau stain to visualize total protein loading and use for protein normalization. Images were captured using a standard camera phone and membranes were rinsed with DI water until the liquid ran clear. The membranes were blocked with 5% BSA in TBS for 2 h before being probed with primary antibody at a 1:1,000 dilution in 5% BSA in TBS overnight, rinsed with 1% BSA in TBST, and then immunoprobed with horseradish peroxidase–conjugated secondary antibody at a 1:10,000 dilution in 1% BSA in TBST. Antibodies used for probing the proteins of interest were SLN (Millipore, ABT13), PLB (SAB4502212), SERCA1a (Abcam, ab105172), SERCA2a (Abcam, ab2861), UCP1 (Sigma, U6382), and secondary (Thermo Fisher 32460). Protein–antibody complexes were detected using SuperSignal West Pico PLUS Chemiluminescence Substrate (Thermo Fisher 34579). Blots were reused for probing with SERCA1a and SERCA2a and SLN and PLB being probed on the same blot following the Abcam mild stripping protocol. Blots were incubated with two rounds of mild stripping buffer (15 g glycine, 1 g SDS, 10 mL Tween 20, and pH 2.2 in 1 L) for 10 min, followed by two 10 min washes with PBS (1 L of 10× 80 g sodium chloride, 36.3 g sodium phosphate dibasic, 2.4 g potassium phosphate, adjust to 7.4 pH), 2–5 min washes with TBST, followed by blocking in 5% BSA and probed as described previously. Protein bands were visualized by exposing for 2–5 min using an Amersham Imager (AI680UV). Whole blot images are presented in Supporting Material.

### Blood analysis

High-throughput metabolomics analysis was performed at the University of Colorado School of Medicine Metabolomics Facility. AGS plasma samples were thawed on ice and metabolites were extracted by adding chilled 5:3:2 methanol:acetonitrile:water (v/v/v) to each tube, 1:24 plasma:buffer (v/v), followed by subsequent vortexing and centrifugation both at 4°C as described by Nemkov et al. (2019). All supernatants were analyzed twice (20 µL injections) by ultra-high performance liquid chromatography using a Thermo Vanquish UHPLC coupled with a Thermo Q Exactive mass spectrometer in positive and negative polarity modes. For each method, the UHPLC utilized a C18 column at a flow rate of 0.45 mL/min for 5 min.

Data were analyzed using Maven (1.4.20-dev-772), and quality controls were maintained as described by Nemkov et al. (2017).

### Statistical analysis

Quantification of band density in Western blots was analyzed using ImageJ (FIJI). All values were normalized to the densitometric sum of bands visualized through Ponceau staining. The values are presented as box and whiskers. Significance was set at *p* ≤ 0.05 and all densitometry comparisons were performed using a one-way ANOVA with a Tukey’s *post hoc* analysis in the Prism software. When analyzing the blood metabolites, early hibernation was used as the reference time point to normalize and determine fold change and *p*-value. Raw metabolites were filtered by *p*-value (*p* < 0.05), and this subset of data was analyzed for pathway enrichment using the MetaboAnalyst software and the KEGG 2019 database (Kanehisa, 2000; 2019; Kanehisa et al., 2023). All enrichment data are presented using Holms’ *p*-value < 0.05 and representative plots of key significant metabolites.

## Results

### Changes in metabolic indicators

**FIGURE 2:**
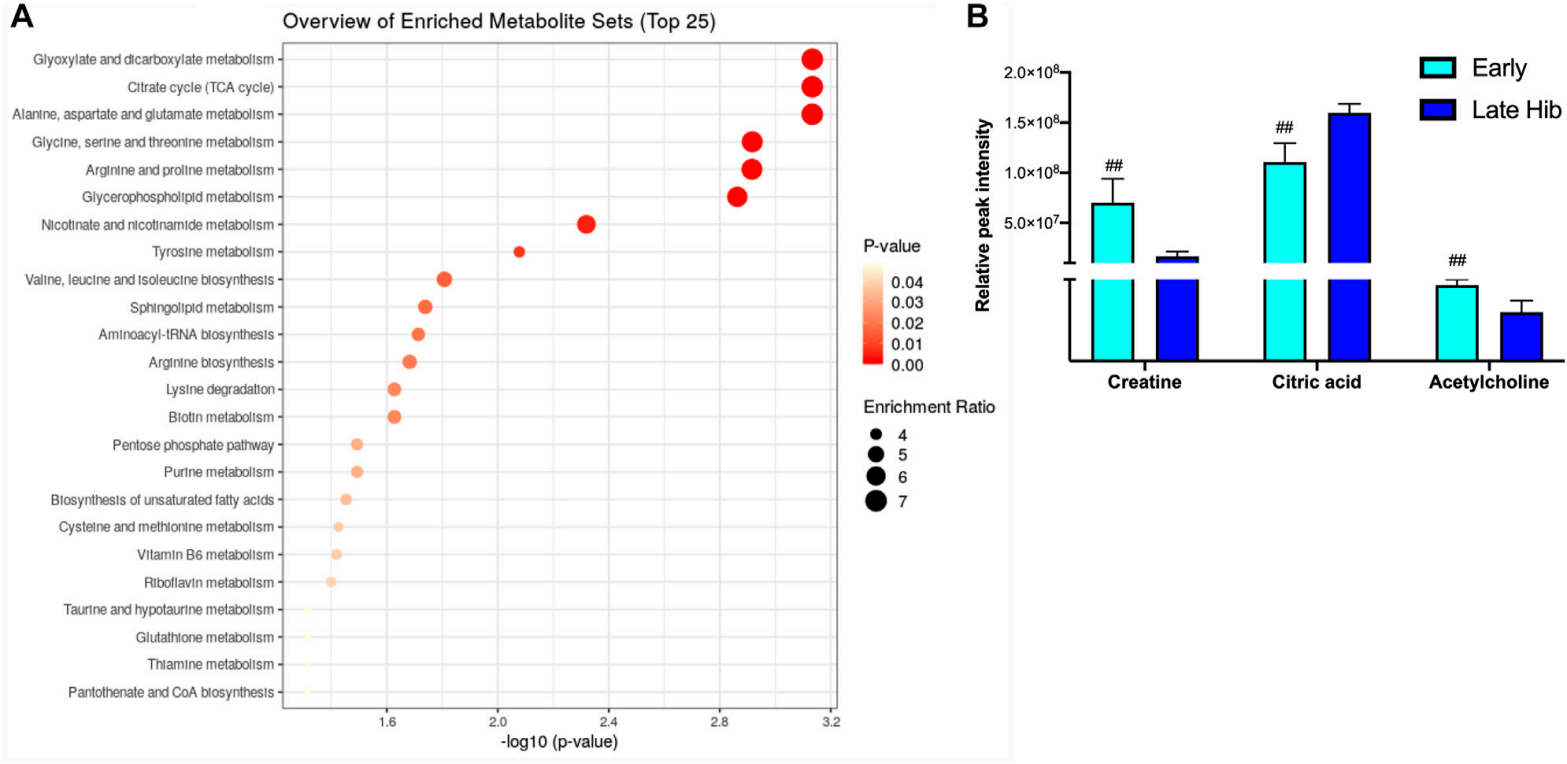
Enrichment analysis of metabolites at two time points during hibernation. **(A)** Late hibernation had energy pathways linked to glyoxylate and citric acid cycles enriched when compared to early (*p* < 0.05). **(B)** Creatine, citric acid, and acetylcholine were three metabolites that were identified as key metabolites influencing the enrichment of pathways between early and late hibernation (*p* < 0.05). Values assessed with MetaboAnalyst enrichment analysis with KEGG 2019 (Kanehisa, 2000; 2019; Kanehisa et al., 2023) as the comparative library and significance threshold of Holms' *p*-value < 0.05.

**FIGURE 3:**
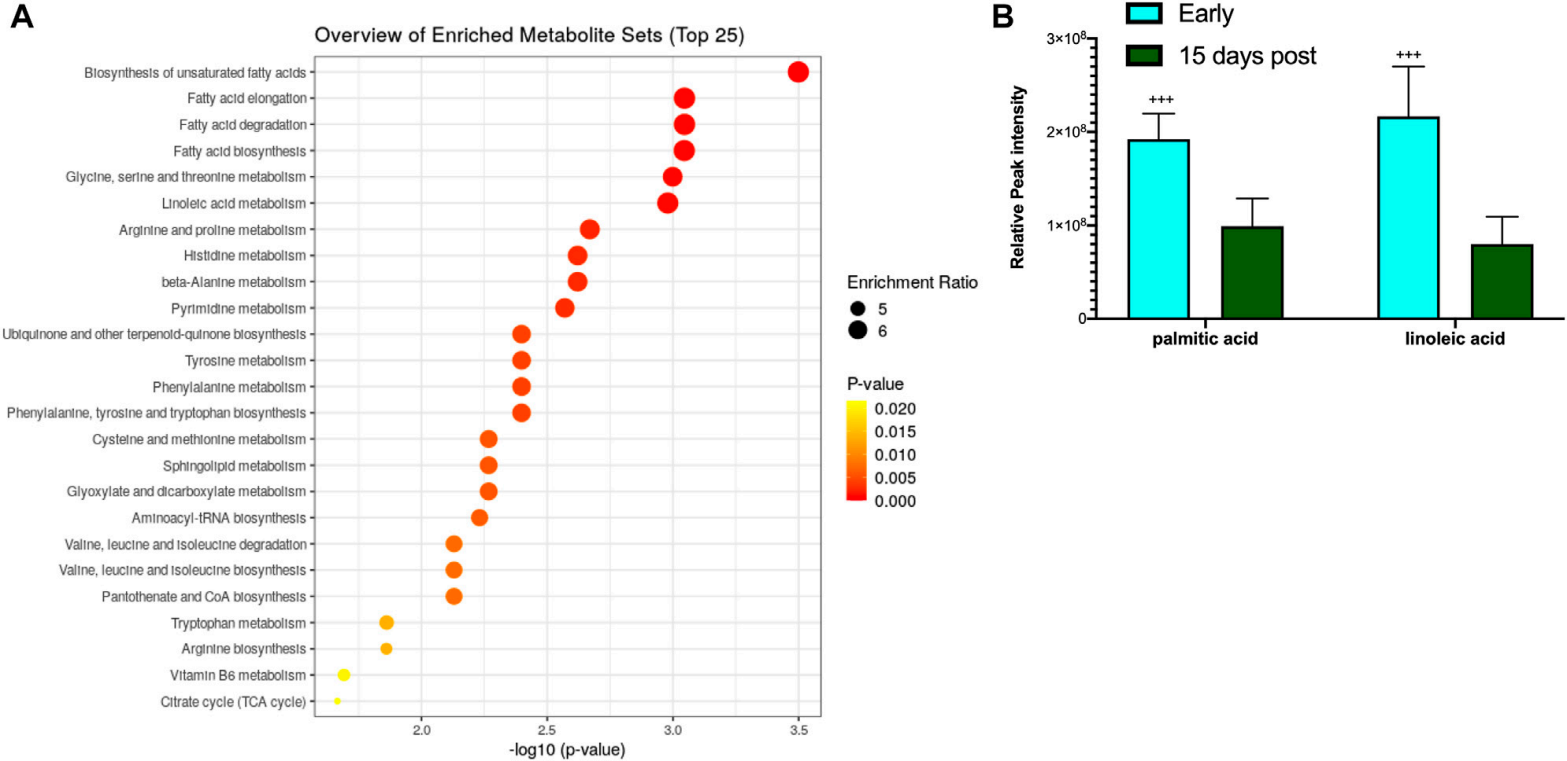
Enrichment analysis of metabolites during hibernation *vs.* terminal arousal. **(A)** Early hibernation compared to 15 days post hibernation showing fatty acid pathways linked to biosynthesis (saturated and unsaturated), elongation, degradation, and linoleic acid metabolism to be enriched during early hibernation (*p* < 0.05). **(B)** Palmitic and linoleic acids are two metabolites that were significantly increased for multiple fatty acid utilization pathways during early hibernation (*p* < 0.05). Values assessed with MetaboAnalyst enrichment analysis with KEGG 2019 (Kanehisa, 2000; 2019; Kanehisa et al., 2023) as the comparative library and significance threshold of Holms' *p*-value <0.05.

The blood metabolite analysis was done on the plasma of AGSs as indicators of full-body metabolic processes and tissue crosstalk. Early/late hibernation and 15 days post hibernation were chosen for enrichment analysis as these were the main inflection points in the protein data. As expected, there were distinct changes in the metabolic pathways related to fatty acid metabolism and usage such as the biosynthesis of fatty acids, elongation, degradation, and linoleic acid metabolism all being enriched during early hibernation as compared to 15 days post hibernation (Figure 2A). The one non-fatty acid pathway enriched in the 15 days post hibernation group when compared to the early hibernation group was glycine/serine/threonine metabolism. Two metabolites with an altered expression between early and 15 days post hibernation and implicated in multiple pathways were palmitic acid and linoleic acid (*p* < 0.05, Figure 2B). The comparison between early and late hibernation had two pathways of enrichment, namely, the citrate cycle and glyoxylate/dicarboxylate that were increased during late hibernation (Figure 3A). The key metabolites involved in these processes, which had significant changes, were creatine, citric acid, and acetylcholine (Figure 3B).

### Fluctuations of UCP1 expression and beiging of white adipose tissue

**FIGURE 4:**
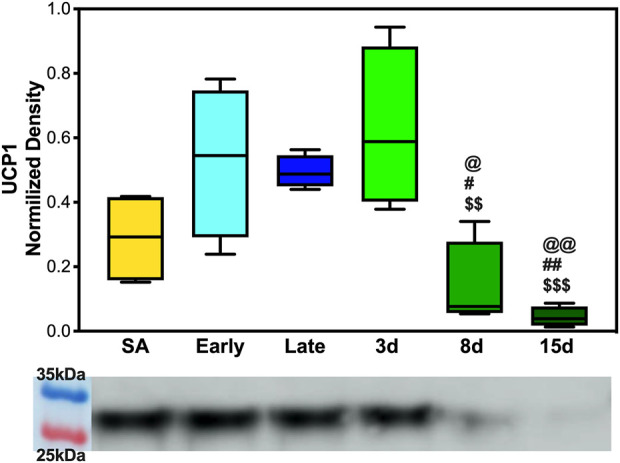
UCP1 expression in brown adipose tissue. (A) Early, late, and 3 days post hibernation had an increased UCP1 expression when compared to 15 days post hibernation (*p* < 0.001, *p* < 0.01, *p* < 0.001) and early, late, and 3 days post hibernation had greater expression than 8 days post hibernation (*p* < 0.05, *p* < 0.05, and *p* < 0.01). Symbols indicate a significant difference based on a one-way ANOVA with Tukey's *post hoc* analysis, with significance set at *p* ≤ 0.05. Symbols indicate significance as follows: &, SA; @, early; #, late; $, 3 days; *, 8 days; +, 15 days. All values are represented as box and whiskers.

To determine whether UCP1 expression fluctuated across the hibernation season and if beiging is naturally induced to increase thermogenic capacity, 5 μg of BAT and WAT protein extracts were analyzed. We observed the upregulation of UCP1 in BAT during early, late, and 3 days post hibernation when compared to 8 days and 15 days post hibernation with a pattern of gradual decrease in expression after terminal arousal (Figure 4). Specifically, the 8 days post arousal UCP1 expression was downregulated fourfold when compared to hibernation, and 15 days post downregulation reached a 10-fold decrease (Figure 4). However, there was no difference in the expression of UCP1 in WAT across all time points as it appears that AGSs hibernating at 2°C do not express UCP1 in WAT during natural hibernation (Supplementary Figure S3).

### Expression of SERCA regulators: SLN

As expected, based on the literature of SLN expression in other species and muscles predominant in fiber type 1, EDL neither showed any levels of SLN expression at any of the time points collected nor were there detectable levels of monomeric or pentameric PLB at any of our collection time points for the EDL muscles (Supplementary Figure S6). However, the DIA, a mixed fiber type constitutively active muscle, showed detectable levels of both proteins. SLN showed a decrease in expression during early hibernation when compared to 8 and 15 days post hibernation with a fold change between 2 and 3 (*p* < 0.05, Figure 3A). Only the monomeric form of PLB was detectable in the DIA but was not analyzable (Supplementary Figure S5).

### SERCA1a and 2a expression in skeletal muscle

To analyze SERCA1a and SERCA2a expressions in AGS, 5 μg of DIA and EDL tissue homogenate was analyzed. SERCA1a showed a depressed expression in AGS diaphragm during late hibernation when compared to summer active, 3 days post, and 8 days post arousal (Figure 6A). Summer active had a twofold increase in the expression compared to late hibernation (*p* < 0.05) and the 3 and 8 days post samples had approximately 2.5-fold increase compared to the late hibernation time point (*p* < 0.01). SERCA2a had a decreased expression at the following time points: summer active, early, late, and 3 days when compared to 8 days post hibernation with the greatest depression in expression during early hibernation (Figure 6C). Summer active, late, and 3 days post hibernation had between 1.6- and 2-fold decrease when compared to 8 days post hibernation (*p* < 0.05). Although early hibernation had a 2.7-fold decrease in expression when compared to 8 days post (*p* < 0.01), both SERCA1a and SERCA2a had similar patterns of expression in DIA with downregulation occurring during early and late hibernation and upregulation after terminal arousal.

The assessment of SERCA1a in EDL did not reveal any significant differences in expression across hibernation at any time point (Figure 6B). However, the expression of SERCA2a did increase in summer active when compared to late hibernation and all post-arousal sample sets (Figure 6D). Summer active had twofold upregulation when compared to the late hibernation time point (*p* < 0.001) and between a 2.3- and 2.6-fold increase in expression over the post hibernation time points (*p* < 0.001). Early hibernation had a similar pattern of expression when compared to that of summer active but the comparison to late, 3 days, and 8 days post hibernation did not reveal any results of statistical significance. However, when compared to 15 days post hibernation, early hibernation had a twofold increase in SERCA2a expression (*p* < 0.05).

## Discussion

It has been well established that the protein–protein interaction between SLN and SERCA1a/2a lowers Ca^2+^ reuptake (v_max_) of SERCA for calcium transport and PLB alters the calcium-binding affinity to SERCA (Bhupathy et al., 2007; Sahoo et al., 2013). It has been demonstrated in model species (mice and rats) that when challenged with cold, SLN and UCP1 both contribute to maintain metabolism and appropriate body composition when functioning alone (Bal et al., 2012; 2017). Studies have shown that in modified mouse models, interruption of both SLN and UCP1 utilization at the same time can be detrimental to defending body temperature and maintaining fat stores (Rowland et al., 2015b; Pant et al., 2016; Bal et al., 2017). Analyzing these proteins in a novel unmodified model that is naturally adapted to both defending and suppressing thermogenic pathways can provide a new insight into their metabolic capabilities; in particular, how the expression of these proteins during periods of metabolic intensity can indicate the use of SERCA uncoupling to regulate thermogenesis and provide an insight into its thermoregulatory relationship and interplay with other heat production pathways. In this study, we set out to examine the expression of proteins key to non-shivering thermogenesis in both muscle and adipose tissues in AGSs after experiencing an IBA. We hypothesized that during the late and early hibernation season, our proteins of interest, namely, SERCA1a/2a, SLN, PLB, and UCP1, would be upregulated in AGS as this would coincide with the largest temperature fluctuations, and thus the metabolic demands would be the greatest. We also hypothesized that the expression of proteins affiliated with muscle uncoupling would have distinct expression patterns based on the muscle sampled.

**FIGURE 5:**
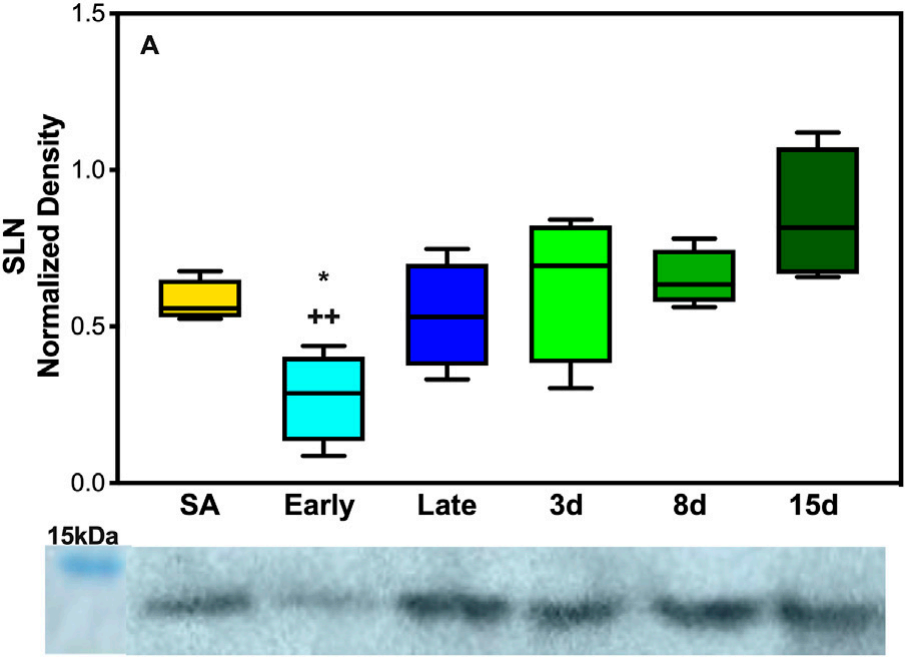
Expression of SERCA regulator SLN in AGS skeletal muscle. **(A)** Expression of SLN in AGS diaphragm shows a decrease in expression during early hibernation when compared to 8 (*p* < 0.05) and 15 days post hibernation (*p* < 0.01). Symbols indicate a significant difference based on a one-way ANOVA with Tukey's *post hoc* analysis, with significance set at *p* ≤ 0.05. Symbols are as follows: *, 8 days; and +, 15 days. All values are represented as box and whiskers.

WAT did not show any expression of UCP1 in contrast to what we hypothesized based on cold exposure studies in other rodent species (Supplementary Figure S3). Beiging of WAT and the accompanying expression of UCP1 in mice were observed during extreme cold and, in some cases, taking as little as 4 h at 4°C to become detectable in the transcriptome (Liang et al., 2019). For AGS, wild burrow temperature can range from 4 to 26°C with the average burrow temperature during the hibernation season being −8°C (Barnes & Buck, 2000). Animals in this project were housed at a constant 2°C, and while this is in the range of the natural burrow temperature, it is well above the average seen in the wild. This may indicate that the standard laboratory hibernaculum temperature was insufficient to induce WAT beiging in AGS. In line with our hypothesis, UCP1 in BAT experienced the largest degree of fold change in expression during early, late, and 3 days post hibernation with downregulation occurring after terminal arousal at 8 and 15 days post hibernation (Figure 4). This is consistent with research in another small mammalian hibernator, the 13-lined ground squirrel, which shows utilization of UCP1 uncoupling is the greatest during torpor and IBA in the hibernation season, when thermogenic stress is at its peak, and lowest during the spring season (Ballinger et al., 2016). This is also supported in Djungarian hamsters where BAT depots are at the greatest size—up to 5% of their body mass—at the end of the summer season and smallest during terminal arousal in spring with BAT atrophy showing a decrease in mitochondrial abundance (Rafael et al., 1985; Hindle & Martin, 2014). Increased UCP1 expression during hibernation is also supported by our metabolomics data that show increased fatty acid metabolism and transport during early hibernation and a decrease during 15 days post hibernation (Figure 5A). UCP1 mitochondrial metabolism is dependent on ß-oxidation, which relies on the metabolism of fatty acids and it is likely that UCP1 expression would be high in conjunction with fatty acid metabolism and utilization (Hankir, 2018). However, the two fatty acids most differentiated between these two time points were linoleic and palmitic acid (Figure 5B). Palmitic acid is not an ideal fuel source in mouse models and has been shown to not feed directly into ß-oxidation in brown adipocytes (Vergnes et al., 2011; Hankir, 2018). Although linoleic and palmitic acids are not optimal fuel sources, they may be recruited into the lipid membrane allowing for modification in the conformation of membrane-bound proteins (Beck et al., 2006; 2007). UCP1 is an integral membrane protein and has been shown to activate exclusively in the presence of long-chain fatty acids, with palmitic acid and linoleic acid enrichment in lipid bilayers increasing UCP1 activation and conductance (Beck et al., 2006; 2007). Therefore, it is likely that enrichment of these fatty acids may indicate possible mediation of bilayer conformation changes in UCP1 in hibernating AGS allowing for increased UCP1 functionality and overall heat production during periods of increased metabolism rather than increases in ß-oxidation. This could also affect the function of SERCA, another integral membrane protein that depends heavily on bilayer composition for functionality. It has been shown that in the hearts of hibernating Syrian hamsters that increased linoleic acid in the sarcoplasmic reticulum membrane allowed animals to reach a lower body temperature and increase SERCA activity during entrance into and during torpor (Giroud et al., 2013). This is supported by our results of linoleic acid being lower during periods of low metabolic demand when compared to the hibernation season when SERCA activity may be more heavily relied on to maintain thermoregulation in AGS.

In EDL, there was no observed difference in SERCA1a expression at any point during the hibernation season but the alternative isoform, SERCA2a, had observed changes in expression. SERCA2a protein increased during the active season when compared to the collection points during hibernation (Figures 2B,D). Additionally, the uncoupler SLN showed no expression in EDL at any of the time points in this study. This was not in line with our hypothesis, but a possible explanation could be the fiber type specificity and preferential fuel utilization during hibernation. In rats, EDL is a type II dominant muscle with 95–98% type II and 2–5% type I muscle fibres (Eddinger et al., 1985; Soukup et al., 2002; Schiaffino & Reggiani, 2011). Type I muscle fibers are characterized by using fatty acid fuel sources, resistance to fatigue (long-duration activity), recruitment for shivering, SLN expression, and SERCA2a expression. By contrast, type II muscle fibers are known for glycolytic fuel utilization, fast twitch (utilized for strong fast movements), less efficient shivering, lack of SLN content, and SERCA1a expression (Close, 1967; Minamisawa et al., 2003; Babu et al., 2005; Babu et al., 2007). However, type II fibers can be subdivided into A, B, and X with type IIa being similar to I, resistant to fatigue and more oxidative, whereas type IIx fibers have moderate oxidative and glycolytic capacity, and type IIb fibers have a high glycolytic capacity and high fatigability (Schiaffino & Reggiani, 2011).

In rodents, fiber type switching during certain environmental stressors is possible. For example, a predominant type II fiber type muscle such as EDL could start to express alternative fibers, potentially due to the demand or available fuel sources. During hibernation, fatty acid oxidation and utilization are the main sources of thermogenic fuel which may encourage fiber type switching to type IIa predominance during the hibernation season. The downregulation of SERCA2a during late and post hibernation runs counter to this postulation. However, this could be due to the hybridization of type I fibers with type IIa fibers and a reliance on isoform switching to type IIa fibers in EDL to balance two functions during hibernation and post hibernation: one is to utilize fatty acid metabolism more efficiently during times of resource scarcity in early and late hibernation (Figure 5A) and the other is to prepare for the rigors of the active season upon emergence in the spring when animals are grazing, mating, evading predators, and in general having higher activity levels that may require fast explosive movements that are dependent on type II fibers. The expression of SERCA1a and SERCA2a likely provides limited information on fiber type predominance and switching in EDL during hibernation, and future fiber typing in AGS could add greatly to these results.

In contrast to EDL, DIA had decreased expression levels of SERCA1a and SERCA2a during late and early hibernation and similar patterns of expression in SLN during early hibernation (Figures 2A,C, 3). DIA in rodents is a mixed fiber type muscle with approximately 50:50 fiber type makeup (Eddinger et al., 1985). During torpor, respiratory frequency is between 2–6 breaths/min and increases to 160–180 breaths/min during an IBA (Tøien et al., 2001; Christian et al., 2014). Both SERCA1a and SERCA2a in DIA had similar decreased expression patterns during early and late time points which can be explained by 1) more efficient utilization of fuel resources and 2) the activity level of the diaphragm being greatly decreased during torpor when compared to an IBA or during terminal arousal. All samples were collected during arousal, and it is possible that SERCA1a and SERCA2a can have further depressed expressions during torpor *vs.* IBA, and these results can be impacted by the sampling method employed in this study.

The response of SLN was the opposite of what was initially hypothesized with the lowest levels of expression in the DIA occurring during early hibernation and was not detectable in the EDL (Figure 3, Supplementary Figure S6). SLN uncoupling may not be required in early hibernation as UCP1 uncoupling in BAT may be sufficient in maintaining body temperature, and the addition of shivering provides a sufficient thermogenic resource for successful IBAs. By contrast, after terminal arousal when fat stores have been depleted, fatty acid utilization is decreased and UCP1 expression lowers (Figure 4). SLN uncoupling may be required to defend body temperature until animals can roam free of burrows and use ambulatory movements for heat production. In theory, AGSs utilize SLN uncoupling during periods of peak metabolic activity and thermogenic stress. For an AGS, this would be during the process of arousal when body temperature rises from −2.9°C to 40°C (Barnes & Ritter, 2019). Our animals were sampled while fully aroused, when peak metabolic activity had elapsed, and normothermic temperature had been achieved, which could be masking some of the occurring muscle NST. Alternatively, these results can indicate that SLN may be performing a role outside uncoupling during the terminal arousal. In mouse SLN overexpression models, SLN has been shown to increase the endurance of muscles, increase resistance to fatigue, and prime the muscles in response to metabolic demand without switching fiber types (Sopariwala et al., 2015). Increased levels of SLN expression in AGS 8 and 15 days post hibernation(Figure 3A) can be explained by this phenomenon and supports our results as DIA activity greatly increases during terminal arousal and SLN can provide a stop-gap measure to reduce the fatigability of the muscle until fiber type dominance can compensate for increased activity going into the active spring and summer months.

**FIGURE 6:**
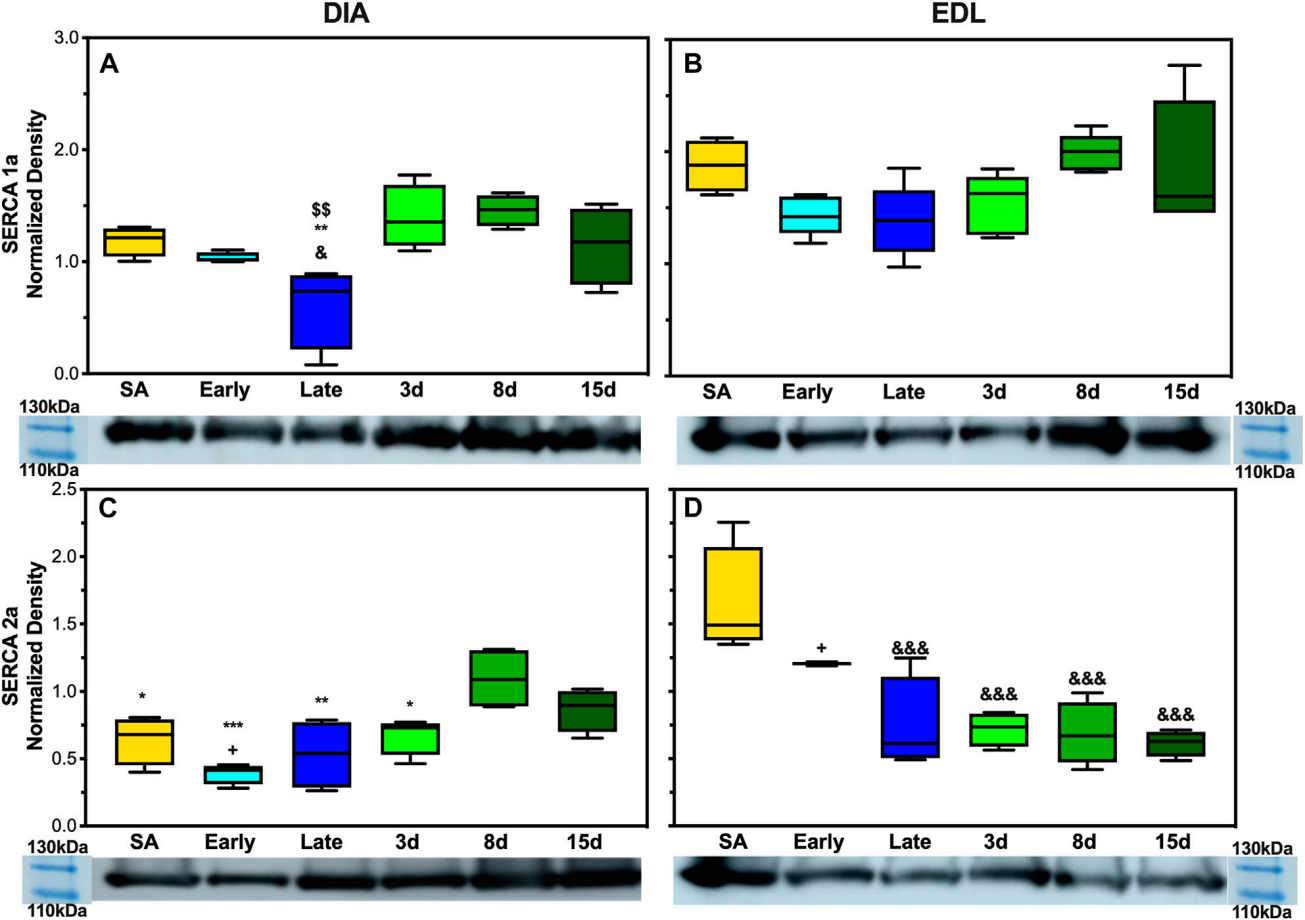
Expression of SERCA1a and SERCA2a in AGS skeletal muscle. **(A)** Expression of SERCA1a in AGS diaphragm shows a decrease in expression during late hibernation when compared to SA (*p* < 0.05), 3 days post hibernation (*p* < 0.01), and 8 days post hibernation (*p* < 0.01) **(B)** SERCA1a was not detectably different at any time point in EDL. **(C)** SERCA2a shows an increased expression in the diaphragm during 8 days post hibernation when compared to summer active (*p* < 0.05), early (*p* < 0.001), late (*p* < 0.01), and 3 days post hibernation (*p* < 0.05). 15 days post hibernation was increased when compared to early hibernation (*p* < 0.05). **(D)** SERCA2a in EDL had a decrease in expression at late hibernation (*p* < 0.001), 3 days post hibernation (*p* < 0.001), 8 days post hibernation (*p* < 0.001), and 15 days post hibernation (*p* < 0.001) when compared to SA. 15 days post hibernation had decreased when compared to early hibernation (*p* < 0.05). Symbols indicate a significant difference based on a one-way ANOVA with Tukey's *post hoc* analysis, with significance set at *p* ≤ 0.05. Symbols are as follows: &, SA; @, early; #, late; $, 3 days; *, 8 days; +, 15 days. All values are represented as box and whiskers.

Taken together, these results are in line with previous data that muscle groups show specificity in the expression of SLN and SERCA isoforms (Eddinger et al., 1985; Bal et al., 2021). Both EDL and DIA show distinct patterns of expression in SLN, SERCA1a, and SERCA2a in AGSs (Figure 5; Figure 6, and Supplementary Figure S6). This could allow AGS to recruit specific muscles for thermogenic maintenance and that fiber type makeup may determine the contribution of a singular muscle group to total NST. These data are also supported by the literature in mice with SLN expression occurring only in slow-twitch muscles and negligible in fast-twitch muscles in non-cold acclimated mice (Pant et al., 2015). This indicates that the cold conditions of a standard hibernaculum may not be sufficient in this cold-acclimated species to induce SLN expression in EDL, as seen in neonatal mice, or to induce UCP1 expression in WAT (Pant et al., 2015). Additionally, the results of this study highlight the interplay between BAT and muscle NST. With the interesting phenomenon of decreases in UCP1 expression coinciding with the increases in SLN expression during terminal arousal at 8 and 15 days post hibernation (Figures 4, 5). This is aligned with data that show that when BAT thermogenesis is impaired either by ablation in mice or UCP1^−/−^ rats, reliance on muscle-based thermogenesis is increased (Bal et al., 2016; Warfel et al., 2023). In addition, both of these studies present limitations to muscle NST that can explain our results: one is that animals relying solely on NST cannot maintain body temperature during prolonged periods of cold exposure (Warfel et al., 2023) and the other is that muscle NST is more energetically expensive than BAT and would require exogenous food sources (Bal et al., 2016). Both of these postulations are in line with our results as SLN expression peaks after terminal arousal in AGSs when spring temperatures increase and food sources become more plentiful. This could indicate a more complex synergy between BAT and muscle thermogenesis in AGSs during hibernation and throughout the year as a whole.

## Conclusion

Although this study had a limited sample size, we could show that AGSs differentially express proteins involved in muscle NST based on the muscle group. Specifically, AGSs express the recently discovered SERCA uncoupler SLN, although it still remains unknown to what extent is this pathway recruited. Our data suggest that brown adipose NST is a strongly upregulated process in AGS during hibernation and that SLN is reduced, which could be a mechanism for allowing shivering mechanisms in muscles or to utilize endogenous fuel sources more efficiently. AGSs may utilize SLN uncoupling in the DIA and other tissues not analyzed in this study to maintain thermogenic rheostasis during hibernation. Furthermore, a similar pattern has been shown in 13 lined ground squirrels (Oliver et al., 2019) and the ability to control NST has been suggested to have great evolutionary implications in other species (Grigg et al., 2022). With many pathways involved in hibernation being conserved across mammalian species, understanding the extent of SLN uncoupling and its role in metabolism, energy expenditure, and fuel utilization during hibernation could provide insight into the ability of this conserved pathway to affect energy expenditure in other species, such as humans.

To further define the utilization of SLN uncoupling and expression of patterns of proteins linked to NST in both muscle and adipose, a broader range of skeletal muscle groups with a variety of fiber type makeup can be collected from both aroused and torpid animals and analyzed for the uncoupling pathway expression. Animals can also be housed at lower temperatures to mimic the wild environment to induce more differentiated expression patterns particularly to see if UCP1 expression in WAT and SLN expression in EDL can be induced. Additionally, full-scale fiber typing of the AGS muscle tissue can be done to see if 1) it is consistent with other rodent models and 2) the extent to which fiber type switching is prevalent across the hibernation season and how that incorporates into muscle group contribution to non-shivering muscle thermogenesis and fuel utilization during periods of thermogenic stress (Heinis et al., 2015, Shaikh et al., 2016).

## Data Availability

The raw data supporting the conclusions of this article will be made available by the authors, without undue reservation.
