# Supplementary material for: Non-shivering thermogenesis is differentially regulated during the hibernation season in Arctic ground squirrels
Source: Front Physiol. 2023 Jul 13;14:1207529. doi: 10.3389/fphys.2023.1207529 (PMC10372343; doi:10.3389/fphys.2023.1207529)
Supplement: Supplementary file 1 [file Image1.pdf]

Non-shivering thermogenesis is differentially regulated during hibernation in Arctic Ground Squirrels

Oliver, S.R., M. Johannsen, J. Rogers, M. Hunstiger
Correspondence: Ryan Oliver sryan.oliver@gmail.com

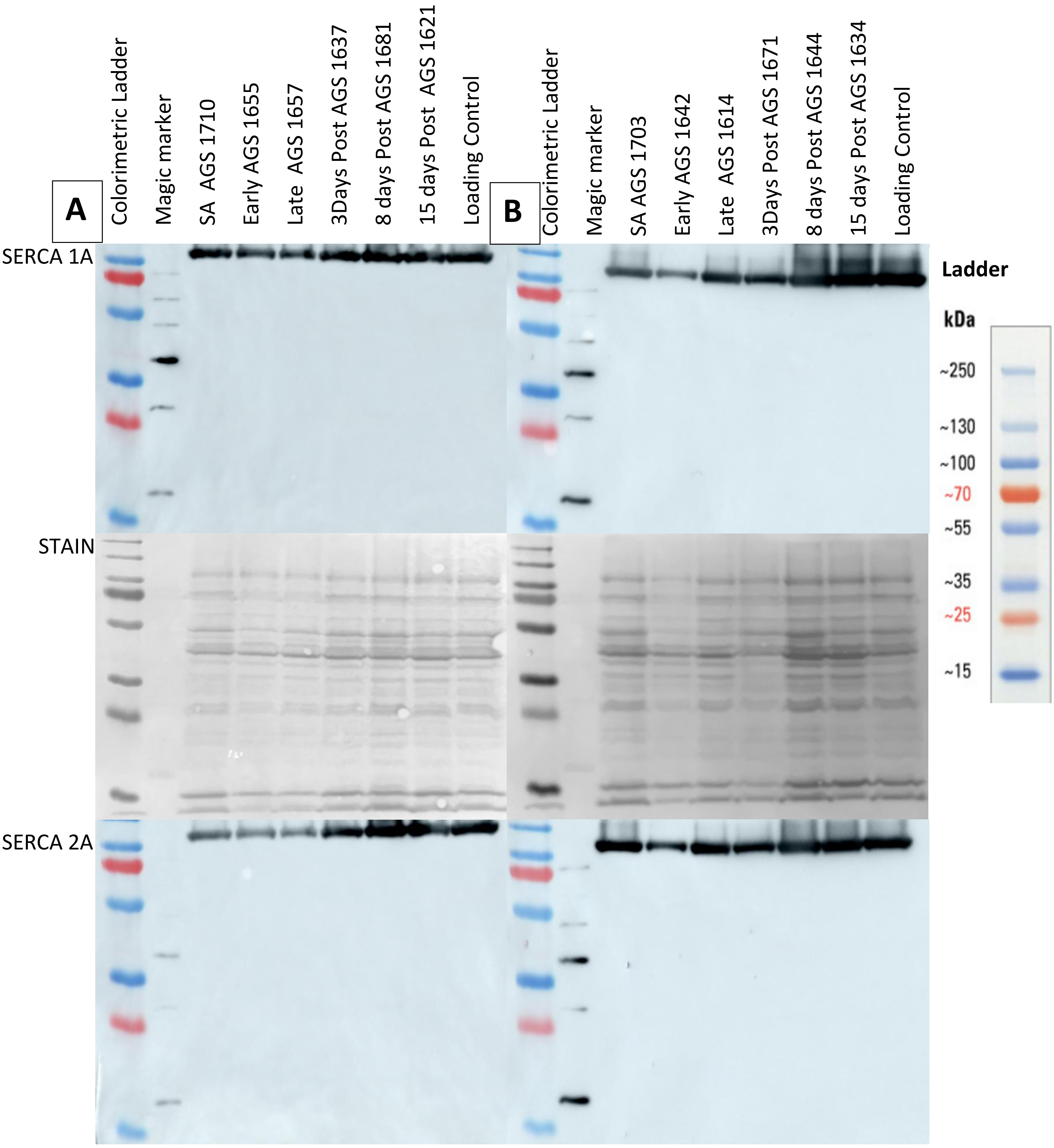

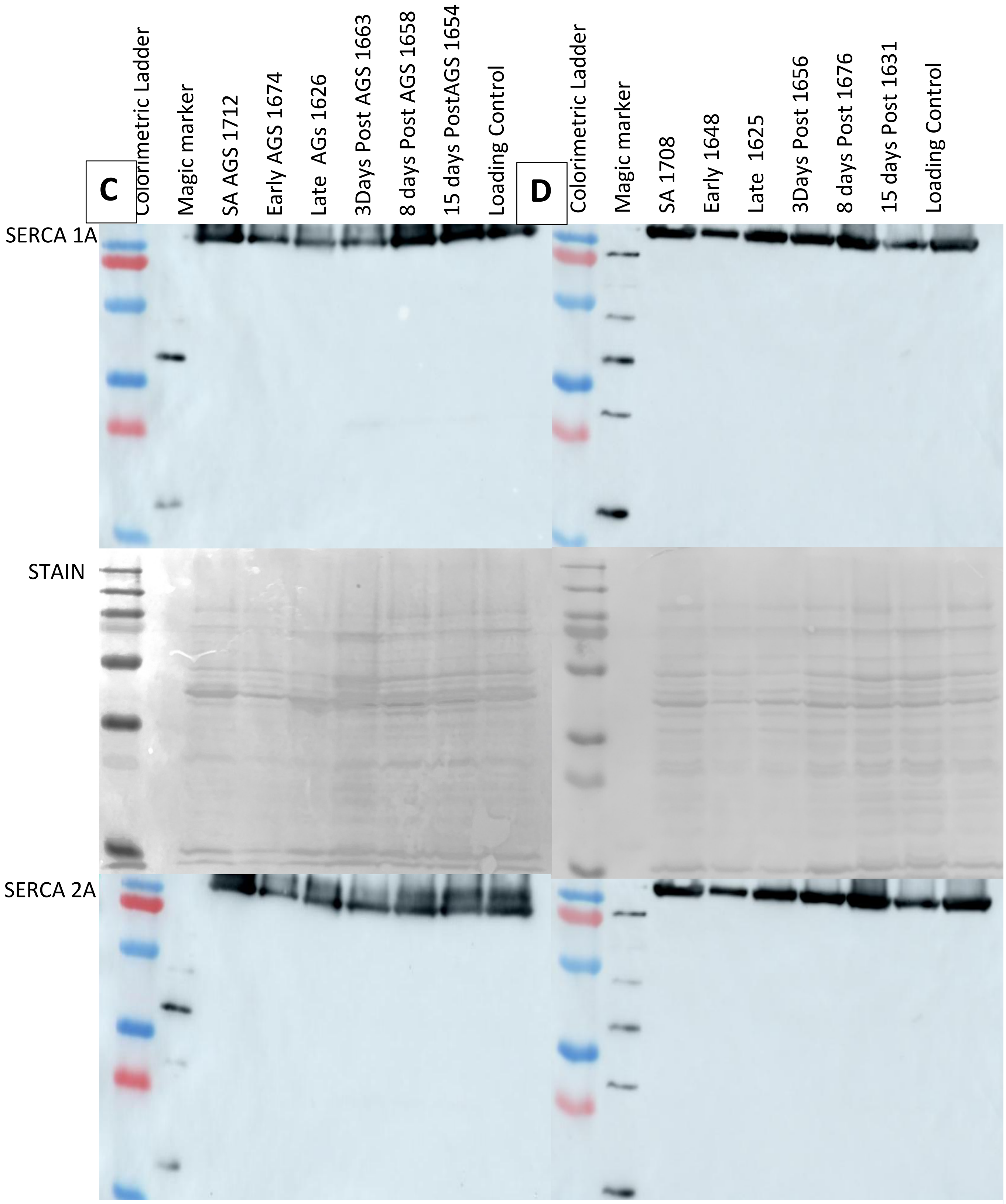

Supplemental F1. Diaphragm Raw SERCA Blots. Panels A-D show all four sample sets for the SERCA 1a and 2a matched with the total protein visualized through ponceau stain. Each panel shows a complete sample set for a combined total of n=4 for each time point with associated labeled colorimetric marker for molecular weight validation of SECRA at 110kDa.

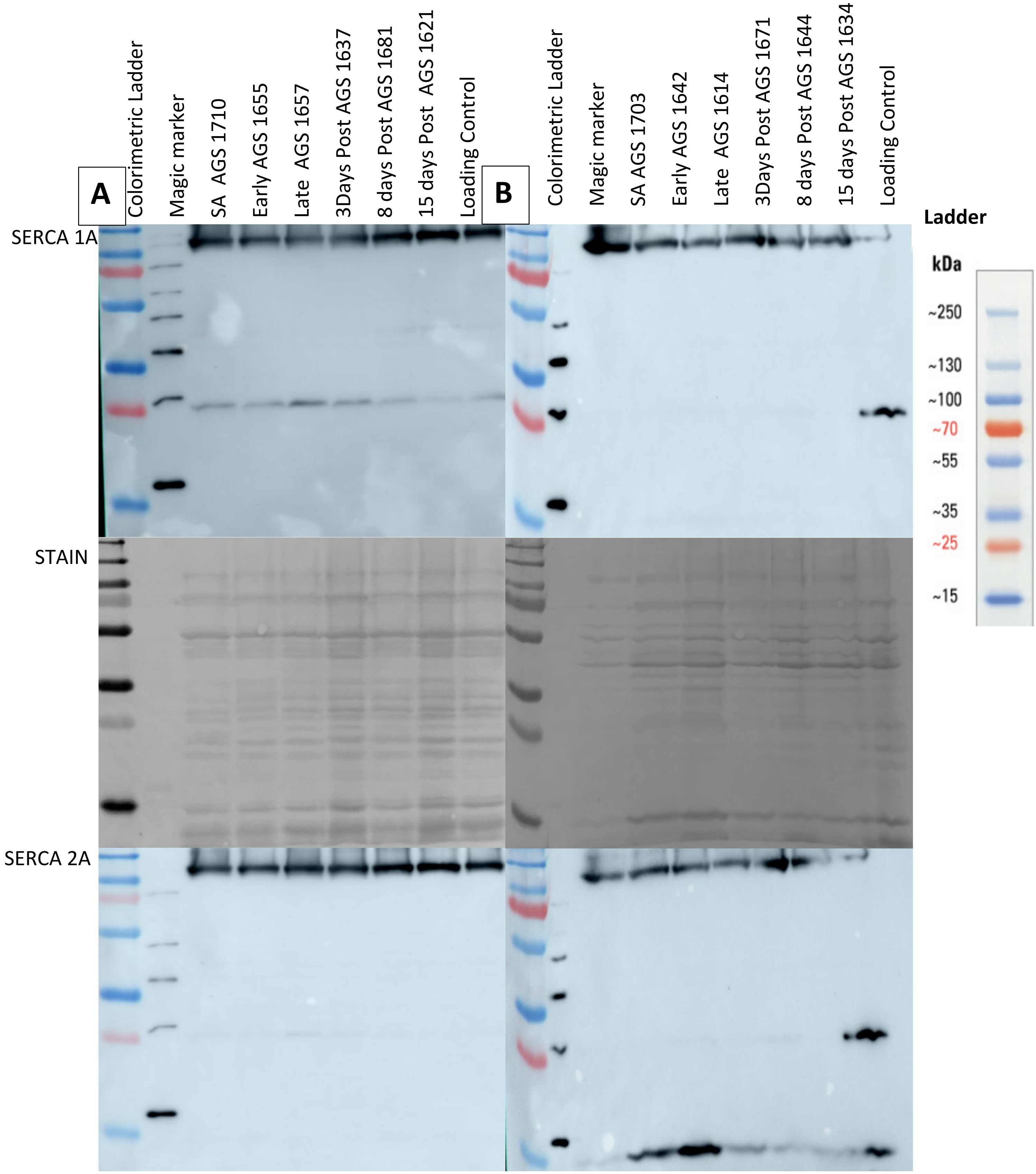
A
B
Colorimetric Ladder
Magic marker
SA AGS 1710
Early AGS 1655
Late AGS 1657
3Days Post AGS 1637
8 days Post AGS 1681
15 days Post AGS 1621
Loading Control
SA AGS 1703
Early AGS 1642
Late AGS 1614
3Days Post AGS 1671
8 days Post AGS 1644
15 days Post AGS 1634
SERCA 1A
STAIN
SERCA 2A
Ladder
kDa
~250
~130
~100
~70
~55
~35
~25
~15

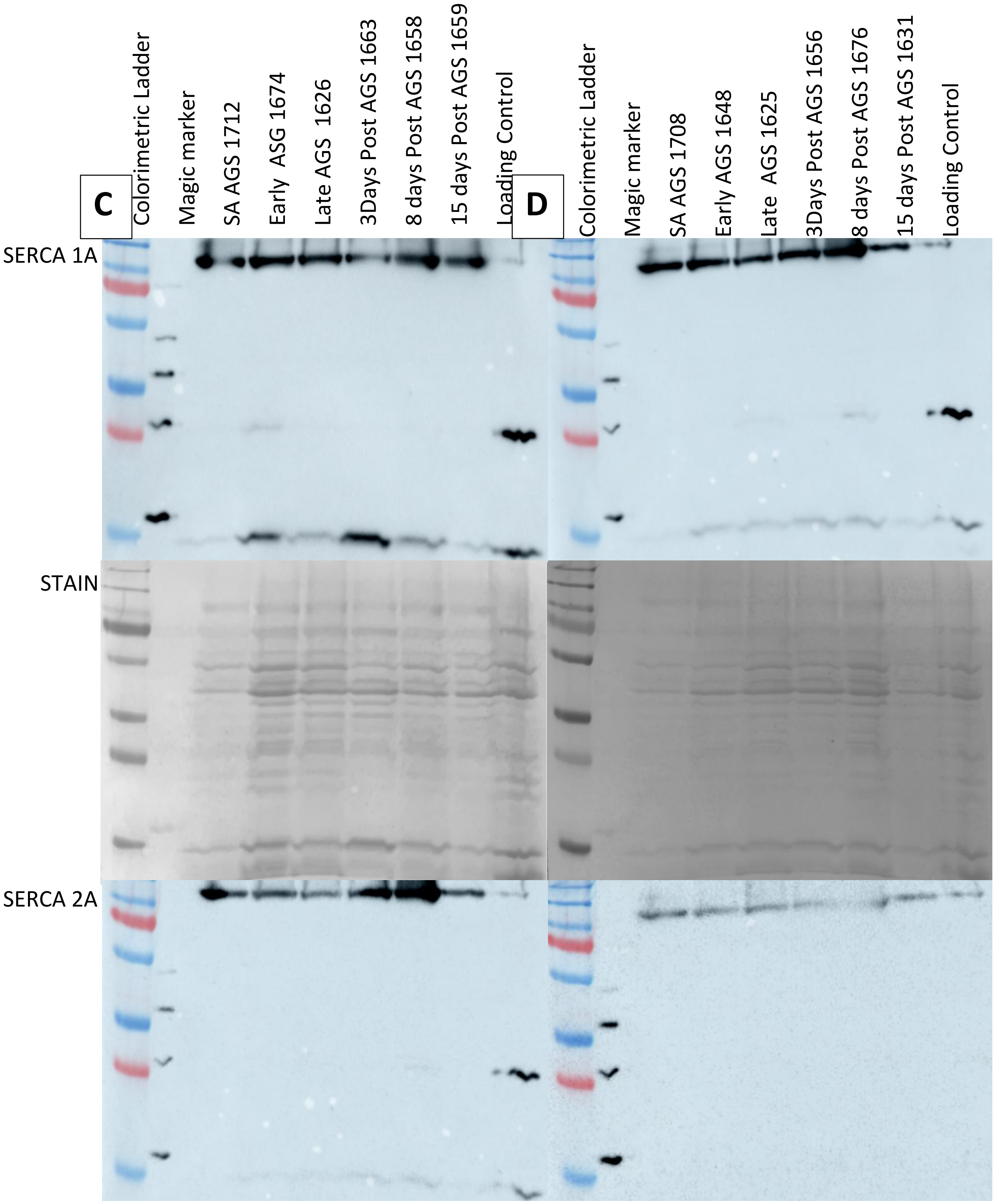

Supplemental F2. EDL Raw SERCA 1a/2a Blots. Each of the panels (A-D) show 1 set of data points pre through post hibernation for a total of n=4. Each blot is matched with total protein image and colorimetric ladder for control measurements and molecular weight authentication of 110Kda.

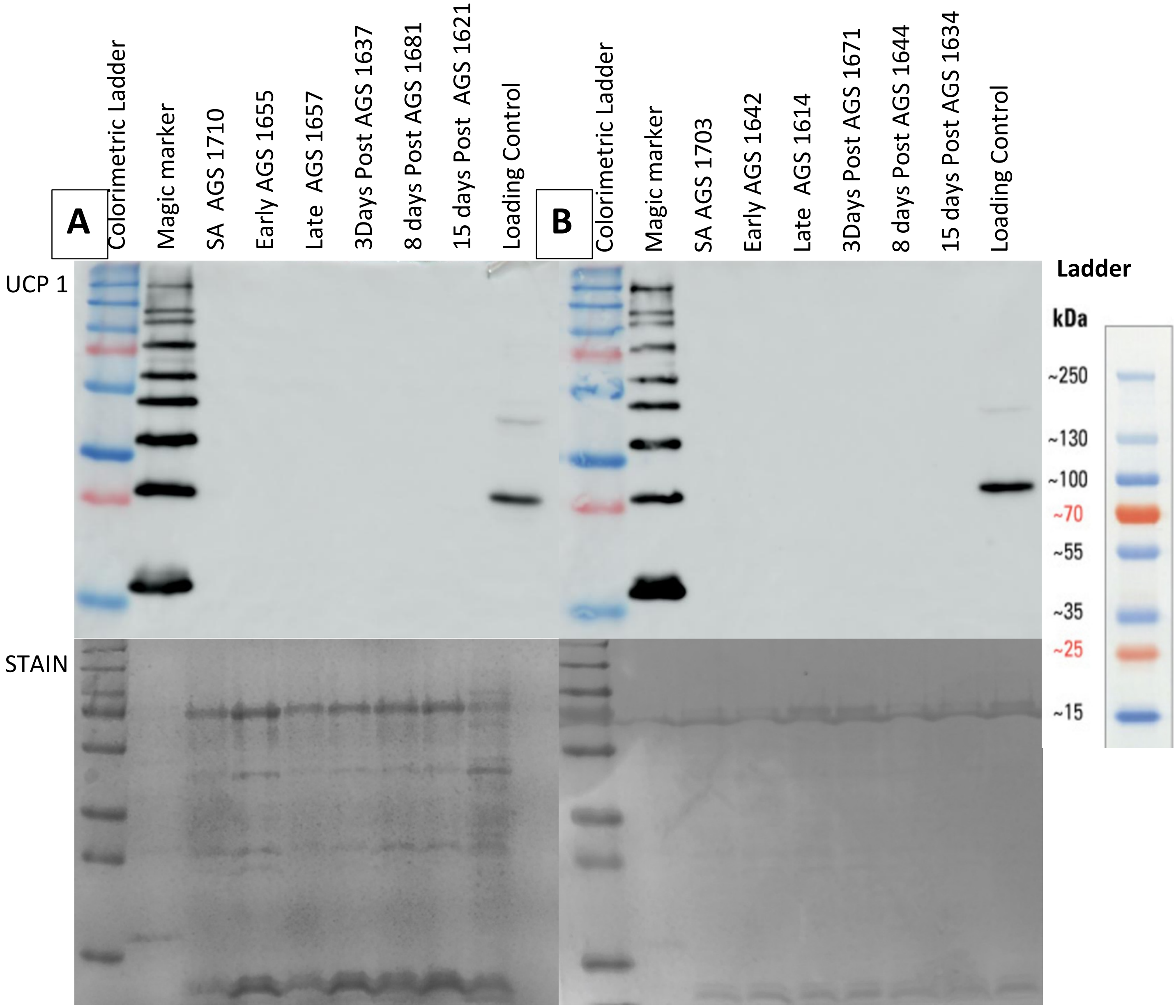
A
Colorimetric Ladder
Magic marker
SA AGS 1710
Early AGS 1655
Late AGS 1657
3Days Post AGS 1637
8 days Post AGS 1681
15 days Post AGS 1621
Loading Control
B
Colorimetric Ladder
Magic marker
SA AGS 1703
Early AGS 1642
Late AGS 1614
3Days Post AGS 1671
8 days Post AGS 1644
15 days Post AGS 1634
Loading Control
UCP 1
STAIN
Ladder
kDa
~250
~130
~100
~70
~55
~35
~25
~15

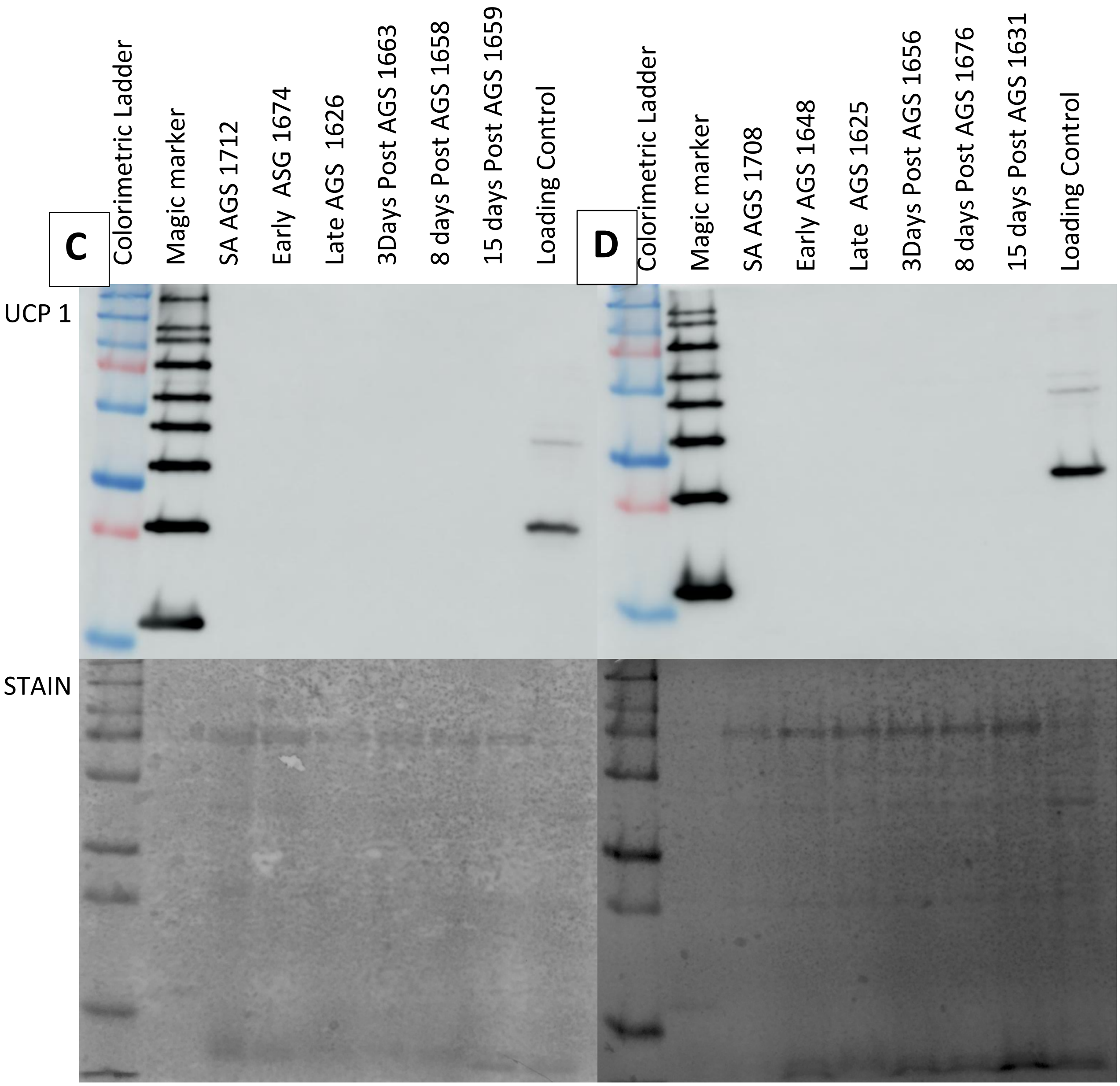

Supplemental F3. White Adipose Raw UCP1 Data. Each of the panels (A-D) show 1 set of data points pre through post hibernation for a total of n=4 for each time point. Each raw blot is matched with its associated ponceau stain for total protein control and validated using a brown adipose control to verify molecular weight against protein ladder. No white adipose samples showed any UCP1 expression at any of the collection time points.

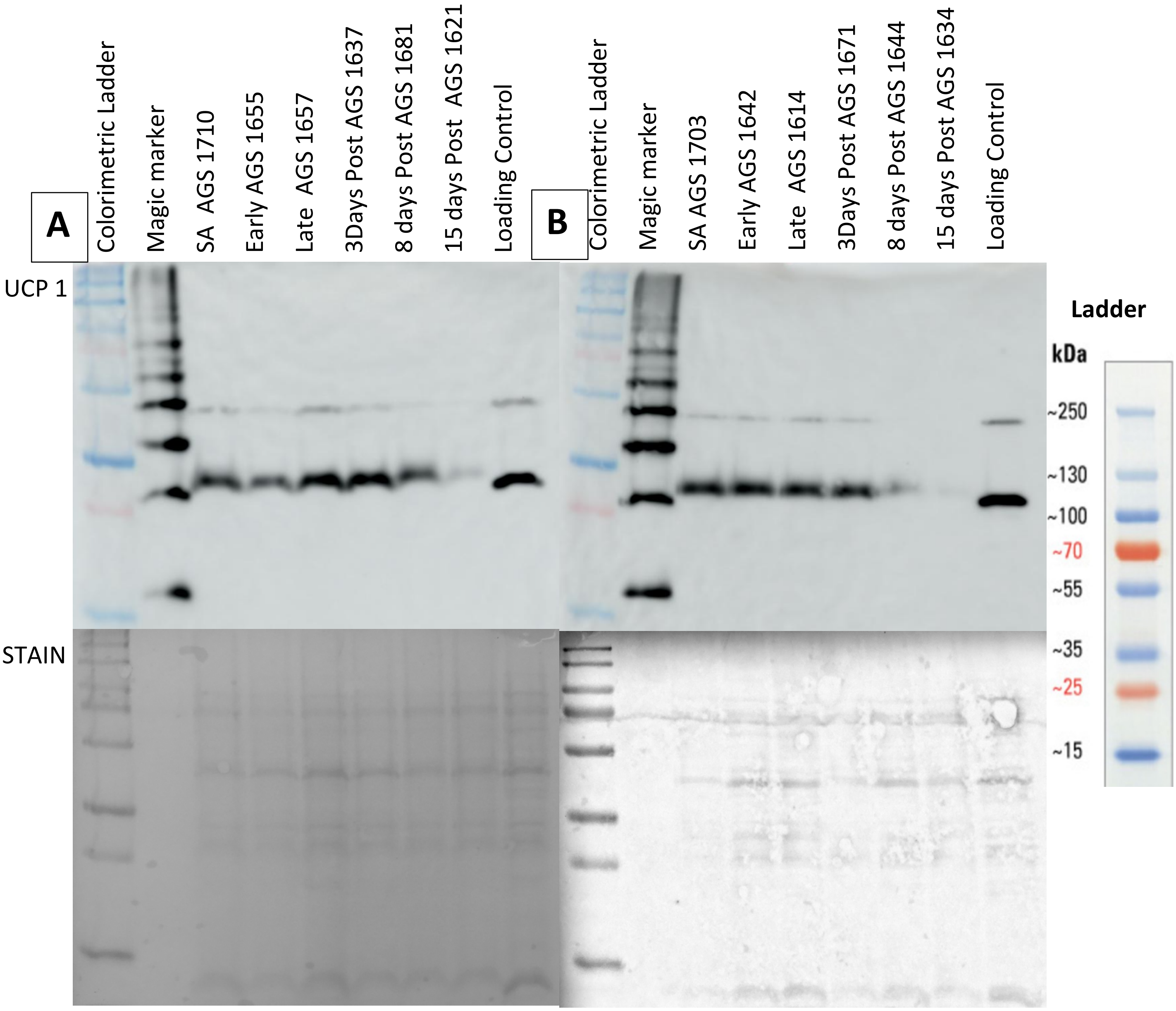

A
Colorimetric Ladder
Magic marker
SA AGS 1710
Early AGS 1655
Late AGS 1657
3Days Post AGS 1637
8 days Post AGS 1681
15 days Post AGS 1621
Loading Control
B
Colorimetric Ladder
Magic marker
SA AGS 1703
Early AGS 1642
Late AGS 1614
3Days Post AGS 1671
8 days Post AGS 1644
15 days Post AGS 1634
Loading Control
UCP 1
STAIN
Ladder
kDa
~250
~130
~100
~70
~55
~35
~25
~15

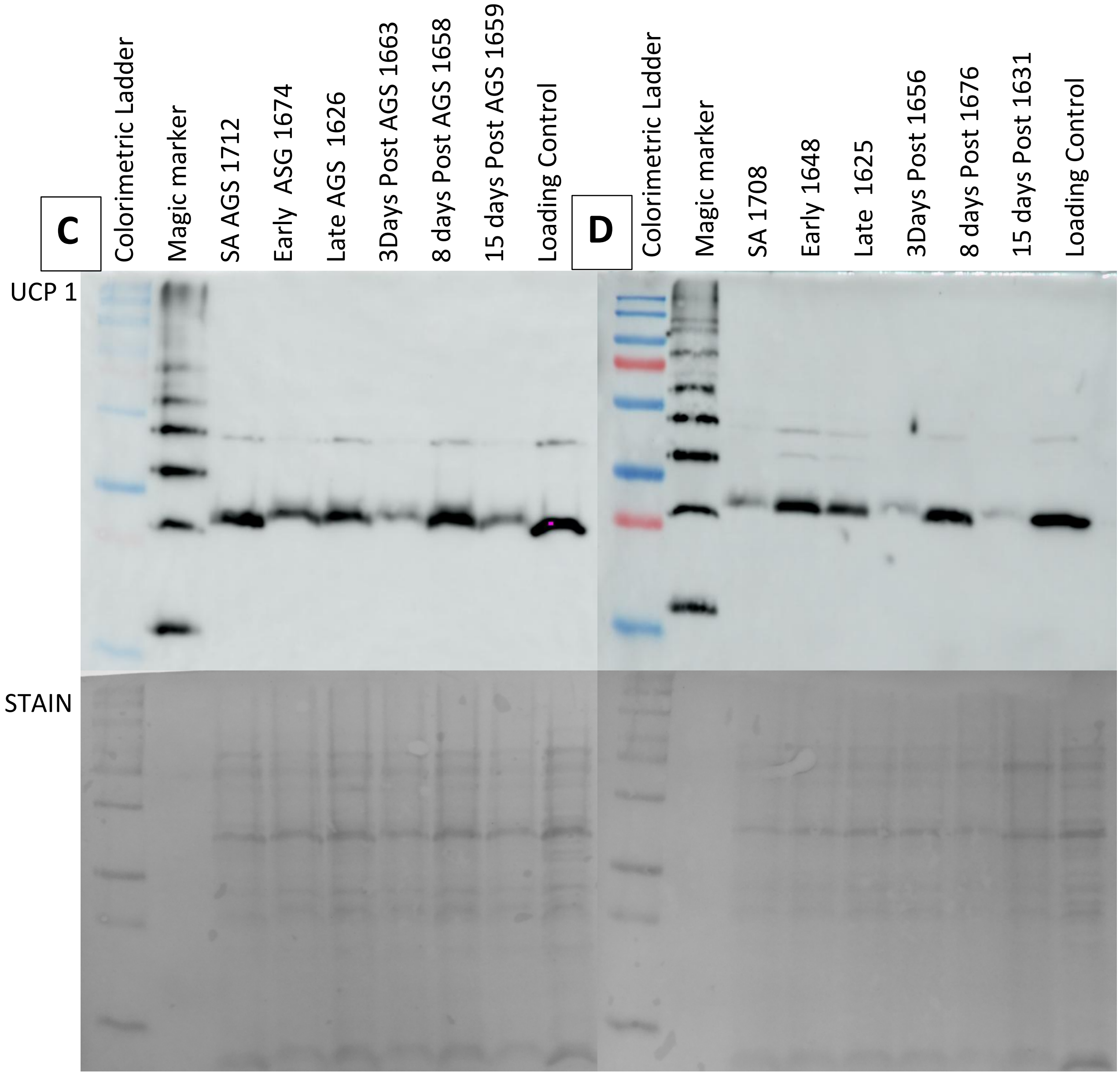

Supplemental F4. Brown Adipose Raw UCP1 Data. Each of the panels (A-D) show 1 set of data points pre through post hibernation for a total of n=4 for each time point. Each raw blot is matched with its associated ponceau stain for total protein control and validated using a brown adipose control to verify molecular weight against protein ladder with UCP1 showing at 33kDa.

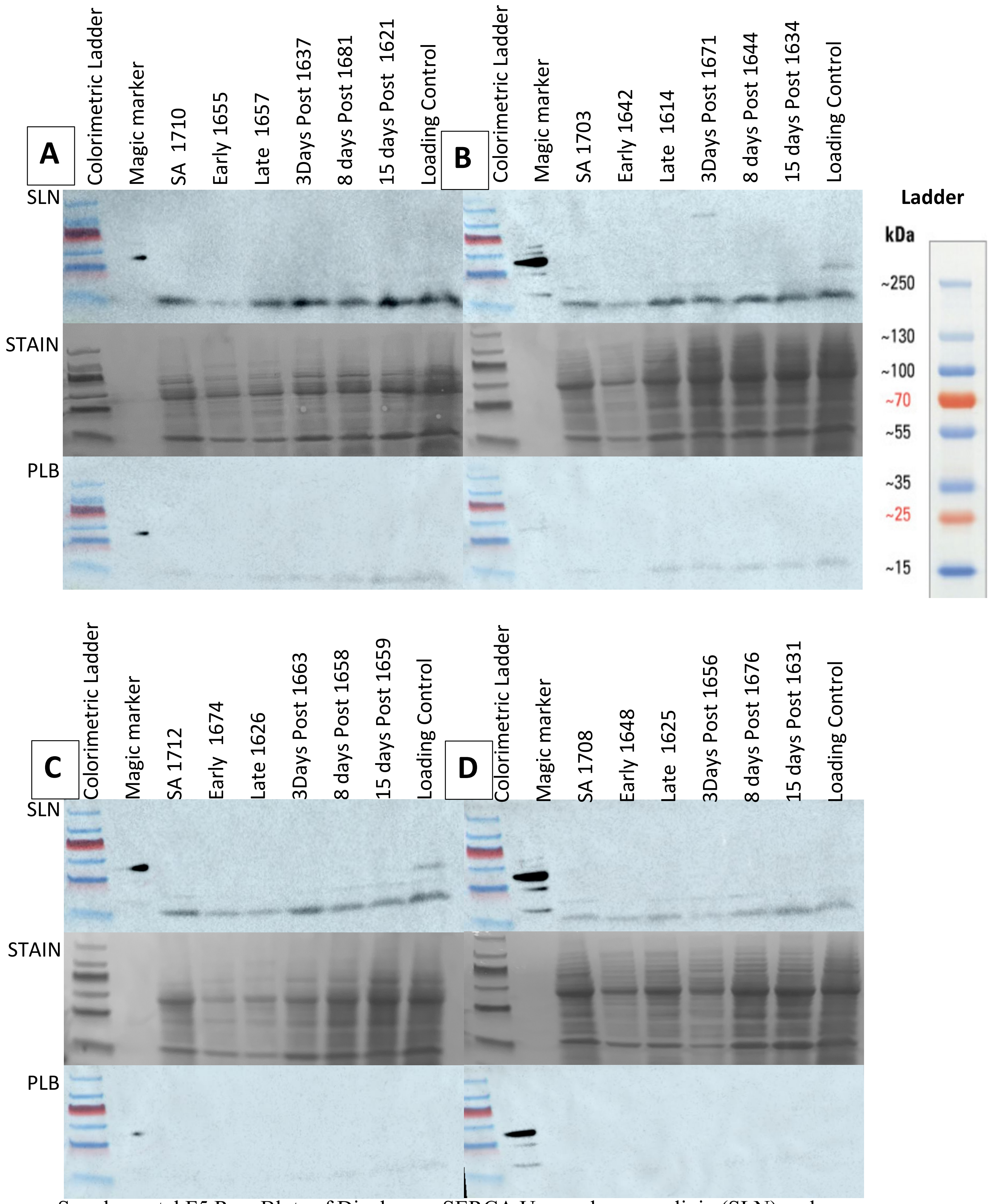

Supplemental F5 Raw Blots of Diaphragm SERCA Uncouplers sarcolipin (SLN) and phospholamban (PLB) Each of the panels (A-D) show 1 set of data points pre through post hibernation for a total of n=4 for each time point. Each blot is matched with total protein image and colorimetric ladder for control measurements and molecular weight authentication of 3kDA and 6kDA for monomeric SLN and PLB and 25Kda for pentameric PLB.

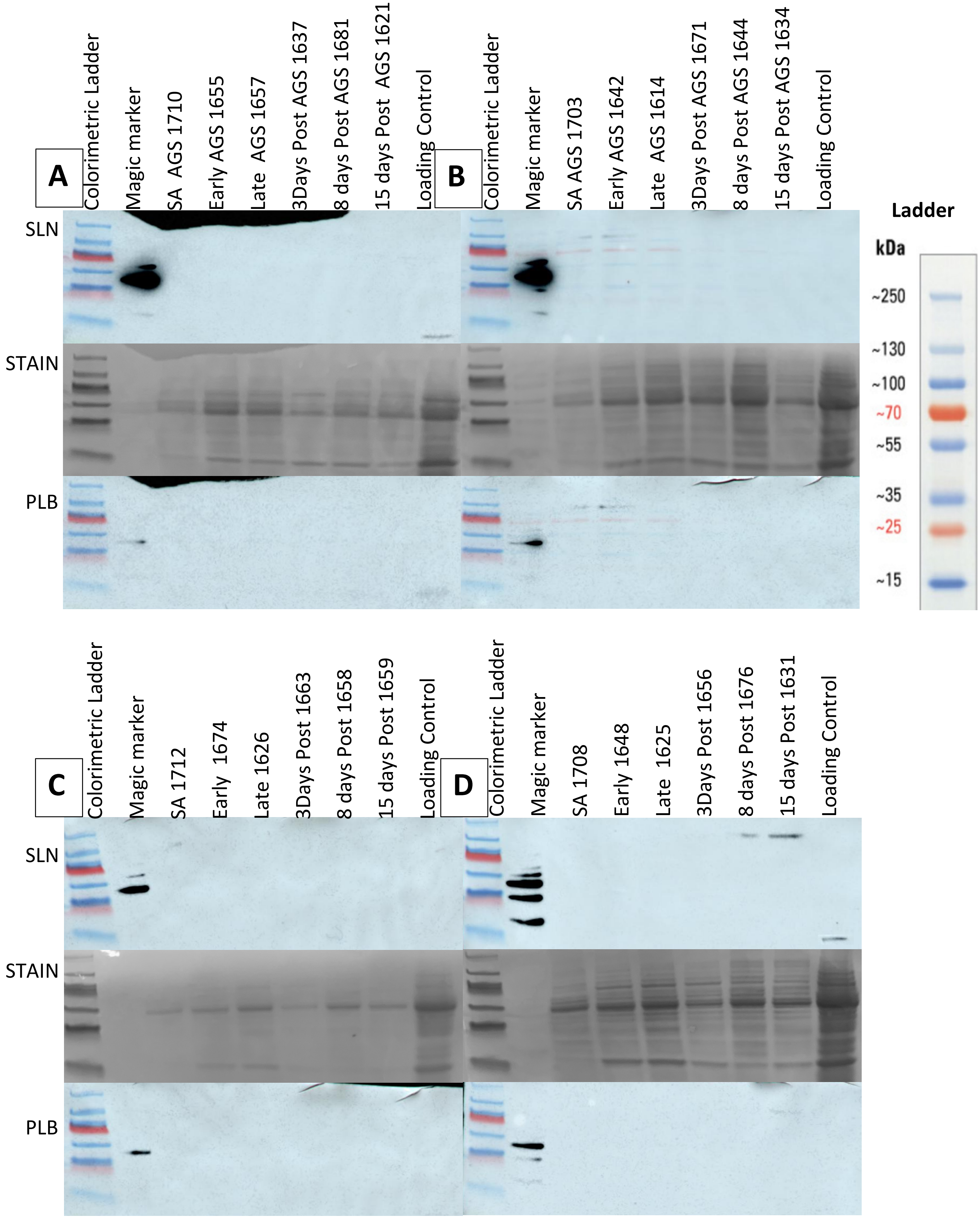

Supplemental F6. Raw Blots EDL SERCA Uncouplers sarcolipin (SLN) and phospholamban (PLB) Each of the panels (A-D) show 1 set of data points pre through post hibernation for a total of n=4 for each time point. Each blot is matched with total protein image and colorimetric ladder for control measurements and molecular weight authentication of 3kDA and 6kDA for monomeric SLN and PLB and 25Kda for pentameric PLB.

	Total Cmpd	Hits	Statistic Q	Expected Q	Raw p	Holm p	FDR
**Biosynthesis of unsaturated fatty acids**	36	3	76.014	11.111	0.00031712	0.010148	0.0055948
**Fatty acid elongation**	39	1	76.664	11.111	0.00089993	0.027898	0.0055948
**Fatty acid degradation**	39	1	76.664	11.111	0.00089993	0.027898	0.0055948
**Fatty acid biosynthesis**	47	2	76.569	11.111	0.0009019	0.027898	0.0055948
**Glycine, serine and threonine metabolism**	33	2	70.481	11.111	0.0010028	0.028078	0.0055948
**Linoleic acid metabolism**	5	1	75.779	11.111	0.001049	0.028324	0.0055948

Supplemental Table 1**.** Raw Enrichment Analysis of Metabolites for Early versus 15- Days Post Arousal. The following include all pathways that after raw p-value adjustment had Holm's p- value <0.5 and thus significantly enriched. Values were calculated using MetaboAnalyst enrichment analysis with Kegg 2019 as the reference library.

	Total Cmpd	Hits	Statistic Q	Expected Q	Raw p	Holm p	FDR
**Glyoxylate and dicarboxylate metabolism**	32	2	77.775	11.111	0.00073494	0.017639	0.0054811
**Citrate cycle (TCA cycle)**	20	1	77.783	11.111	0.00073523	0.017639	0.0054811
**Alanine, aspartate and glutamate metabolism**	28	1	77.783	11.111	0.00073523	0.017639	0.0054811
**Glycine, serine and threonine metabolism**	33	5	74.874	11.111	0.0012105	0.025421	0.0054811
**Arginine and proline metabolism**	38	3	74.904	11.111	0.0012135	0.025421	0.0054811
**Glycerophospholipid metabolism**	36	2	71.784	11.111	0.0013703	0.026035	0.0054811

Supplemental Table 2 **.** Raw Enrichment Values of Early versus Late Hibernation. The following include all pathways that after raw p-value adjustment had Holm's p- value <0.5 and thus significantly enriched. Values were calculated using MetaboAnalyst enrichment analysis with Kegg 2019 as the reference library.
